# Melatonin Enhances Photo-Oxidation of 2′,7′-Dichlorodihydrofluorescein by an Antioxidant Reaction That Renders N1-Acetyl-N2-Formyl-5-Methoxykynuramine (AFMK)

**DOI:** 10.1371/journal.pone.0109257

**Published:** 2014-10-02

**Authors:** David Hevia, Juan C. Mayo, Dun-Xian Tan, Aida Rodriguez-Garcia, Rosa M. Sainz

**Affiliations:** 1 Departamento de Morfologia y Biologia Celular, School of Medicine, University of Oviedo, Oviedo, Spain; 2 Instituto Universitario Oncologico del Principado de Asturias (IUOPA), Oviedo, Spain; 3 Department of Cellular and Structural Biology, University of Texas Health Science Center at San Antonio, San Antonio, Texas, United States of America; Instituto de Química, Universidade de São Paulo, Brazil

## Abstract

The indolamine melatonin (MEL) is described as an antioxidant and a free radical scavenger. However occasionally, the indoleamine has been reported to increase free radicals with insufficient mechanistic explanation. In an attempt to find a reason for those controversial results, a potential mechanism that explains MEL prooxidant activity is investigated. The current controversy about redox detection methods has prompted us to search a possible interaction between MEL and dichlorodihydrofluorescein (DCFH_2_), perhaps the most widely fluorescence probe employed for free radicals detection in cellular models. Here, it is demonstrated that melatonin potentiates the photooxidation of DCFH_2_ in a cell-free system, increasing the production of its fluorescent metabolite. Indeed, MEL works as an antioxidant scavenging hydroxyl radicals in this system. Thus, this reaction between MEL and DCFH_2_ produces N1-acetyl-N2-formyl-5-methoxykynuramine (AFMK), a biogenic amine with antioxidant properties too. This reaction is O_2_ and light dependent and it is prevented by antioxidants such as N-acetylcysteine or ascorbic acid. Furthermore, when DCFH_2_ has been employed to evaluate antioxidant or prooxidant activities of MEL in cellular models it is confirmed that it works as an antioxidant but these results can be modulated by light misleading to a prooxidant conclusion. In conclusion, here is demonstrated that DCFH_2_, light and melatonin interact and results obtained using these fluorescence probes in studies with melatonin have to be carefully interpreted.

## Introduction

Oxidative stress has an important impact in human health. Its implication in several disorders including atherosclerosis, diabetes, neurodegeneration or cancer has been widely investigated. The principal components of oxidative stress are a variety of chemical species such as nitric oxide (NO), superoxide anions (O_2_^•−^), hydroxyl radicals (^•^OH) and hydrogen peroxide (H_2_O_2_) among others. Some of these molecules are generated exogenously or produced endogenously from several sources including oxidative phosphorylation in mitochondria. Given its important role in physiology and pathology, there is an increasing interest in developing accurate methods to measure free radical production in cells.

One of the principal drawbacks of oxidative stress research has been the accuracy when measuring ROS production in *in vivo* systems. There are currently several methods developed for measuring free radicals inside cells including chemiluminescence of luminol or lucigenin [1], cytochrome c reduction [2] or ferrous oxidation of xylenol orange [3] as well as some other commercially available fluorescence probes. However, among all of them, 2′,7′-dichlorofluorescein (DCF) staining is by far the most widely employed for the analysis of ROS and cellular oxidative stress [4], [5]. To measure ROS in cells [6], DCFH_2_-DA is used because it can be easily taken up and it is more resistant to oxidation than DCFH_2_. Upon internalization it is rapidly de-acetylated and after that it reacts with ROS to produce a fluorescence product [7]. Given its simplicity and sensitivity, DCFH_2_-DA [4] has been employed to study the production of H_2_O_2_ [8] in several reports by using microplate reader [9] or flow cytometry methods [10].

N-acetyl-5-methoxy-tryptamine or melatonin is an indolamine produced endogenously and secreted into circulation mainly by pineal gland though it is also synthesized in many other locations. In all species studied thus far, its synthesis from tryptophan occurs during darkness [11], [12]. Considering its nocturnal synthesis, melatonin has been linked to sleep promotion [13], a chemical signal of light∶dark cycle [14], and a regulator of reproductive physiology in seasonal breeding mammals among others [15]. Besides regulating circadian and circannual rhythms, melatonin is a major endogenous antioxidant and a free radical scavenger [16]. Melatonin functions as a direct-scavenging molecule and it also stimulates indirectly gene expression and activities of antioxidant enzymes [17]. As a direct scavenger, melatonin reacts with different free radicals including ^•^OH, O_2_^•−^, NO^•^ and alkyl-peroxyl radicals [18]–[20] and indirectly, it stimulates glutathione production and the activities of both, glutathione peroxidase and superoxide dismutase [21], [22]. There is an inverse relationship between melatonin levels and tumour growth, in terms of initiation but also, of progression and metastasis [23]. Although numerous mechanisms have been identified to explain melatonin inhibition of cancer [24], its role as an intracellular redox regulator has been well documented as one of the mechanism by which it could modulate cancer growth [25]. Melatonin has been mostly reported to inhibit cell growth by reducing free radicals production or activity [26] but also, it has been suggested that melatonin by itself promotes cell toxicity and death of some tumour cells through a prooxidant pathway [27]–[30].

Antioxidant and prooxidant activities of melatonin have been previously evaluated by using DCFH_2_ or DCFH_2_-DA staining by other researchers. Furthermore, there are several cases of interactions between DCFH_2_ or DCFH_2_-DA with other molecules. So, a set of experiments to assess any potential interaction between melatonin with DCFH_2_ or DCFH_2_-DA are performed to clarify discrepancies observed about antioxidant or prooxidant properties of the pineal neuroindoleamine when this probe are used.

## Material and Methods

### Chemicals and solutions

2′-7′-dichlorodihydrofluorescein diacetate (DCFH_2_-DA) was purchased from Invitrogen (Life Technologies, Alcobendas, Madrid, Spain). All other chemicals were purchased from Sigma-Aldrich (Tres Cantos, Madrid, Spain). Melatonin (Merck, Darmstadt, Germany) stock (1 M) was prepared in DMSO and then diluted until desired concentration directly in phosphate buffer saline (PBS). Other reagents including catalase (CAT), superoxide dismutase (SOD), ascorbic acid (AA), N-acetyl cysteine (NAC) or H_2_O_2_ were freshly prepared in PBS and used immediately for all assays.

### Light-dark experiments

Light-dark experiments were performed in a hermetic box protected from external light and equipped with a light bulb located at 15 cm from samples. Light used was a 6W linear fluorescent (F6T5/D, GE lighting # 10028) with the following features: Initial Lumen (NOM) 230, Median Lumen (NOM) 185, Colour temperature 6500 K, Nominal initial lumen per Watt (NOM) 38. Other specific parameters such as spectral, power distribution or electric characteristic can be checked at the company web site (www.gelighting.com). Light power reaching samples was 25000 lux. All the experiments were performed at RT. All solutions were placed in open tubes and at the same time for each experiment. Dark experiments were carried out in the same conditions than light experiments but in this case light of box was turned off.

### DCFH_2_ preparation

For cell-free experiments DCFH_2_-DA was deacetilated to DCFH_2_ prior to each experiment following the method described before [31]. Briefly, 0.5 ml DCFH_2_-DA (1.0 mM in methanol) was mixed with 2 ml of NaOH (0.01 M) for 30 minutes at RT. Then, mixture was neutralized by adding 10 ml of NaH_2_PO_4_ (25 mM, pH 7.4). Final solution 1 mM DCFH_2_ was employed within 15 minutes after dilution.

### Fluorescence and absorbance spectroscopy

Absorption spectra of samples containing DCFH_2_-DA or DCFH_2_ in PBS at pH 7.4 with or without MEL, H_2_O_2_, AA, SOD or CAT were measured by using a Cary 50 Bio UV-Vis spectrophotometer (Agilent Technologies, Santa Clara, CA, USA) at room temperature. Changes in absorption were quantified at 501 nm (λ_max_ of DCF).

Fluorescence were measured in quartz cuvettes using a Cary Eclipse fluorimeter (Agilent Technologies, Santa Clara, CA USA) at RT (λ_exc_ = 480 nm, λ_em_ = 500–700 nm). Voltage was set between 400 and 800 V. Since voltage was changed to get enough acquisition, all groups from the same set of experiments were measured at the same time, using the same voltage intensity. For studies under a N_2_ atmosphere, an atmosbag glove bag (Sigma-Aldrich) was used.

### HPLC measurements

HPLC analysis was performed on 1260 Infinity HPLC system (Agilent Technologies, USA) equipped with a binary pump with solvent selection valves, online degasser and a programmable auto-sampler. A tracer Extrasil ODS1 column (250 mm×0.46 mm, 5 µm) (Teknokroma, Barcelona, Spain), operating at 35°C was used. An ODS guard column was placed previously to protect the analytical column. Mobile phase solution was always filtered through a 0.45 µm membrane filter. Identification of the compounds was determined by their retention time (RT) and UV spectrum. All measurements were performed using Chemstation software.

HPLC analysis of MEL, N1-acetyl-N2-formyl-5-methoxykynuramine (AFMK), N1-acetyl-5-methoxykynuramine (AMK) or cyclic 3-hydroxymelatonin (3-COHM) was performed as previously described [32]. Briefly, sodium acetate (20 mM, pH 5.1) in 35% methanol was used as mobile phase. A flow of 0.9 ml/min and different wavelengths (190 at 800 nm) were employed to obtain the spectrum of absorbance for each compound. The elution order was 3-COHM, AMK, MEL and AFMK and absorbance was set at 230/279 nm (absolute/relative maximum) for MEL, 233/380 nm for AMK, 233/337 nm for AFMK and 231/306 for 3COHM. Quantification was performed at 231 nm. Standards of AFMK, AMK and 3COHM were synthesized by using the method reported by Tan et al [33]. Thus, H_2_O_2_ was diluted to 50 mM with PBS (50 mM, pH 7.0) and deferoxamine was dissolved in this solution at a final concentration of 1 mM to chelate any possible trace of free iron. MEL was then added to this solution to make a final concentration of 1 mM. The mixture was incubated for 2 h at RT. The majority components of this solution were then mixed with an equal volume of dichloromethane and shaken horizontally for 10 min. The water phase was discarded and the organic phase was dried under vacuum. The residue was dissolved in a small volume of methanol and fractionated by analytical thin layer chromatography with silica gel on polyester, fluorescent indicator, layer of 250 mm and 20 3 20 cm (TLC) using ethyl acetate as the solvent. The major spot (about 90% in all metabolites), which migrated with an RF of 0.2 (detected with UV lamp at 254 nm) was scraped from the TLC plate and extracted with methanol. The TLC purification was repeated two additional times. The purified product was identified to be AFMK by simple 1H-NMR. For AMK synthesis, the above purified AFMK was dissolved in PBS buffer (50 mM, pH 7.0) at a final concentration of 7 mM and incubated with catalase (2500 U/ml) at room temperature for 24 h. The solution was mixed with two portions of dichloromethane (per volume) and shaken horizontally for 10 min. The water phase was discarded and the organic phase was dried under vacuum. The residue was then dissolved in a small volume of methanol and the enzyme metabolite was fractionated by analytical TLC using ethyl acetate as the solvent. The single metabolite produced by catalase was isolated from TLC plate as described above and identified to be the AMK by 1H-NMR.

DCFH_2_ and DCF were separated by HPLC in an isocratic mode following the method previously reported [34]. A mixture of NaH_2_PO_4_ (20 mM, pH 6.8) and methanol (43∶57) was used as mobile phase. Flow was set at 1 ml/min, at RT and 20 µl of sample were injected. Wavelengths between 190 and 800 nm were used.

HPLC-MS was used to confirm presence of AFMK in samples. Agilent 1290 Infinity (HPLC) and Agilent 6460 triple quad (MS) equipped with a Zorbax Eclipse Plus C18 column (Agilent, 2.1×50 mm, 1.8 µm particle) were used. Mobile phase consisting in two components (A 0.1% formic acid; B ACN with 0.1% formic acid) in gradient mode (5% B to 90% B, 1 to 6 min) with a flow of 250 µl/min at 30°C and 2 µl of injection volume were the optimal parameters chosen. Flow of 5 L/min and temperature of 300°C of nebulization gas was chosen. ESI positive at 3500 V, product ion mode (m/z ion 265 (M+H)^+^) and 10 eV as Collision Energy to fragment precursor ion was used.

### Cell culture experiments

Hippocampal neuronal (HT22) and prostate cancer (PC3) cell lines were cultured in DMEM and DMEM/F12 respectively, supplemented with 10% FBS and 1% antibiotic-antimycotic cocktail. Cells were grown at 37°C in a humidified 5% CO_2_ environment, seeded at a density of 25,000 cell/mL of complete media in 6 or 96 well plates and allowed to attach overnight before experiments. Cells were incubated 24 hours with or without 1 mM MEL. Thereafter, medium was replaced and KRH buffer (50 mM HEPES, 137 mM NaCl, 4.7 mM KCl, 1.85 mM CaCl_2_, 1.3 mM MgSO_4_, 0.1% BSA, pH: 7.4) with 10 µM of 2,7-dichlorofluorescein diacetate (DCFH-DA) was added for 30 min at 37°C in darkness. Fluorescence was measured after 30 min in a microplate reader (λex 485 nm, λem 530 nm - μQuant, Biotek) or from flow cytometer (Beckman-Coulter EPICS-XL Cytometer) as previously described [9], [35].

## Results

### Evaluation of DCFH_2_-DA photooxidation in the presence of melatonin

**Figure 1:**
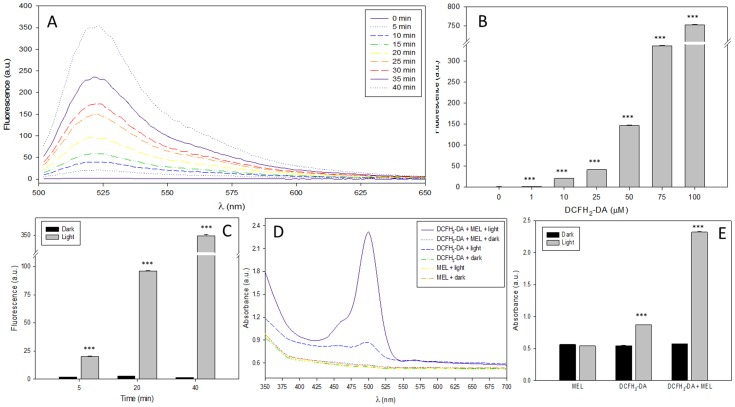
Effect of melatonin on light-induced DCFH_2_-DA oxidation. A) Fluorescence spectrum (λ_exc_ = 485 nm, λ_em_ = 500–700 nm) of DCFH_2_-DA (100 µM) plus MEL (1 mM) under light (0–40 min). B) Fluorescence of several concentrations of DCFH_2_-DA plus MEL (1 mM) and light 60 minutes. ***p<0.001 vs no treat. C) Fluorescence of DCFH_2_-DA plus MEL (1 mM) during 5, 20 or 40 minutes under light or dark conditions. ***p<0.001 vs Darkness. D) Absorbance Spectrum (350–700 nm) of DCFH_2_-DA (100 µM) or MEL (1 mM) alone or mixed under 30 minutes of dark or light conditions. E) Absorbance measurement at 505 nm of MEL (1 mM), DCFH_2_-DA (100 µM), alone or mixed under 30 minutes of light or dark conditions. ***p<0.001 vs Darkness.

DCFH_2_-DA is one of the most widely employed fluorescence probe to measure redox state inside cells. It is a cell permeable precursor of DCFH_2_ that can readily cross membrane. After internalization, it is cleaved by intracellular esterases giving DCFH-DA obtaining DCFH_2_. Therefore, to evaluate a possible interference in the fluorescence of DCF caused by MEL and light reaction, both molecules (DCFH_2_ and DCFH_2_-DA) were employed. Thus, DCFH_2_-DA photooxidation was evaluated by measuring fluorescence emission of its oxidant product in the presence or absence of MEL in both, under light or in darkness. When DCFH_2_-DA was mixed with MEL and exposed to light at different times, a significant increase in fluorescence emission was observed (Fig. 1A). This increase of fluorescence was clearly dependent on time, DCFH_2_-DA concentration (Fig. 1B) and light (Fig. 1C). Similarly, when DCFH_2_-DA alone or plus MEL were exposed to light/dark and absorbance was measured, MEL increased significantly the absorbance of DCFH_2_-DA (Fig. 1D, E).

### Evaluation of DCFH_2_ photooxidation in the presence of melatonin

**Figure 2:**
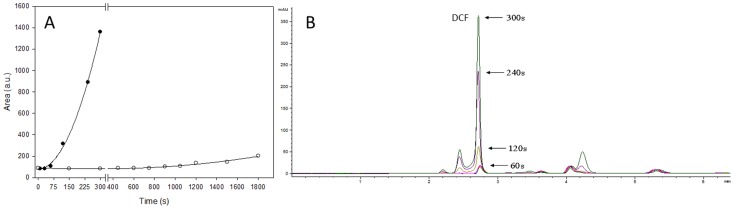
Time-dependence in melatonin effect on DCFH_2_ photooxidation. A) Time course of DCF production by DCFH_2_ (10 µM) alone (-○-) or plus MEL 1 mM (-•-) under light. B) Chromatogram of DCF after DCFH_2_ (10 µM) plus MEL (1 mM) were exposed to light for 60, 120, 240 or 300 seconds.

Once it was observed the enhancement of DCFH_2_-DA photooxidation by MEL, the interaction of DCFH_2_ and MEL was also studied. DCFH_2_-DA was deacetylated to DCFH_2_ which was then mixed with MEL under light. As reported above, a significant increase of time-dependent fluorescence when 100 µM DCFH_2_ was exposed to light was observed. By using 10 µM DCFH_2_ plus 1 mM MEL under light, fluorescence was rapidly increased after few seconds (Fig. 2A). Chromatogram presented in figure 2B showed a production of DCF compound after 60, 120, 240 and 300 seconds plus light and MEL. As shown, after only 60 seconds of exposition to MEL and light, DCF peak is 10 times higher than control.

### Evaluation of DCFH_2_ and DCFH_2_-DA photooxidation in the presence of melatonin under UV light or in a N_2_ atmosphere

**Figure 3:**
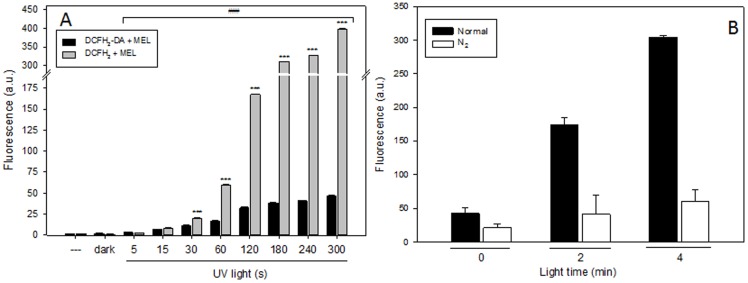
Role of UV light and O_2_ in DCFH_2_-DA and DCFH_2_ photooxidation. A) Fluorescence of DCFH_2_ (10 µM) or DCFH_2_-DA (100 µM) plus MEL (1 mM) under UV light. B) Fluorescence of DCFH_2_ (10 µM) plus MEL (1 mM) under N_2_ or normal atmosphere.

In addition to visible light, UV light was employed to evaluate the photooxidation of DCFH_2_ and DCFH_2_-DA. After DCFH_2_ exposure to UV light, there was an increase in fluorescence, and again, that increase was dependent on time. Similarly to what happens under visible light, when DCFH_2_ was incubated with MEL under UV light, fluorescence emission was significantly higher (Fig. 3A). The increment of fluorescence under UV light is much higher than under visible light since even lower compound concentration gives a much faster time of reaction. The spectrum of fluorescence after light exposure at different times is shown in supplementary material. The increment of fluorescence is 10 times higher when DCFH_2_ was combined with MEL under UV light than when DCFH_2_-DA was employed (Figure S1A). Likewise, MEL was able to increase by 100 fold the fluorescence of DCFH_2_ when they were exposed to UV light for several minutes (Figure S1B).

To check if atmospheric O_2_ has an important role in photooxidation of DCFH_2_ by MEL, an experiment under N_2_ was performed. When O_2_ was eliminated from solution fluorescence did not increase. After 2 minutes under light, fluorescence intensity under N_2_ atmosphere is clearly lower than under normal atmosphere (Fig. 3B). For these experiment it is possible to conclude that O_2_ plays an instrumental role in the photooxidation process.

### Participation of H_2_O_2_ generation by melatonin in DCFH_2_ or DCFH_2_-DA photooxidation

**Figure 4:**
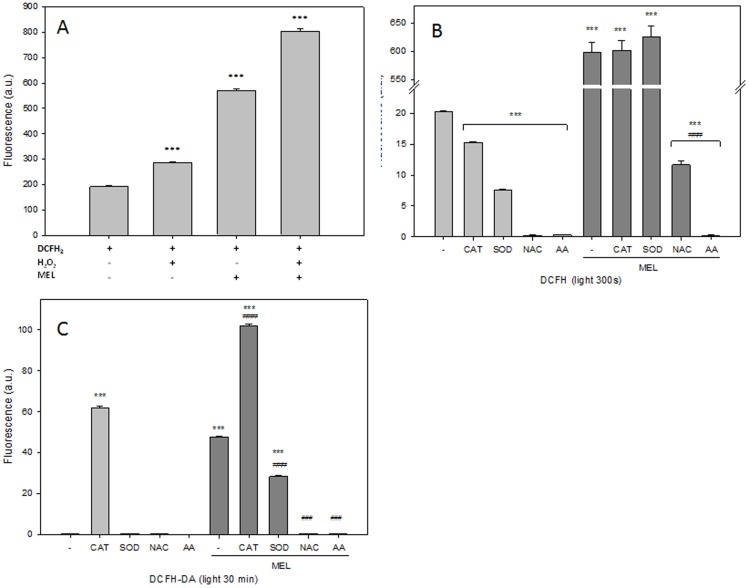
Impact of antioxidants on melatonin enhancement of DCFH_2_ and DCFH_2_-DA photooxidation. A) Fluorescence of DCFH_2_ (10 µM), MEL (1 mM), H_2_O_2_ (10 µM) alone or in combination under light for 300 second. B) Evaluation of fluorescence of DCFH_2_ (10 µM) with CAT (200 U), SOD (200 U), NAC (10 mM) and AA (10 mM) with or without supplementation of MEL (1 mM) under light for 300 seconds. C) Evaluation of fluorescence of DCFH_2_-DA (100 µM) with CAT (200 U), SOD (200 U), NAC (10 mM) and AA (10 mM) with or without supplementation of MEL (1 mM) under 30 min seconds of light exposure.

In order to understand the mechanism of DCFH_2_ photooxidation by MEL, H_2_O_2_ was included in the DCFH_2_ plus MEL mixture solution. After 300 seconds under visible light, the increment of fluorescence was measured. As previously described by others [7], an increment of DCF was observed after either H_2_O_2_ or MEL addition (Fig. 4A). In previous reports [7], [36], [37], the activity of antioxidant enzymes in preventing DCF formation was studied to demonstrate its dependence on ROS production. Consequently, catalase (CAT), superoxide dismutase (SOD), N-acetyl-cysteine (NAC) or ascorbic acid (AA) were employed to inhibit DCF formation after DCFH_2_ or DCFH_2_-DA plus MEL under light. CAT or SOD did not inhibit DCF formation after DCFH_2_ plus MEL exposure under light but they clearly reduced its formation after DCFH_2_ exposure alone (Fig. 4B). On the contrary, antioxidants such as AA or NAC inhibited DCF fluorescence when both DCFH_2_ (Fig. 4B) or DCFH_2_-DA (Fig. 4C) were incubated alone or plus MEL under light [38], [39].

### Production of kynureamines after DCFH_2_ and melatonin reaction

**Figure 5:**
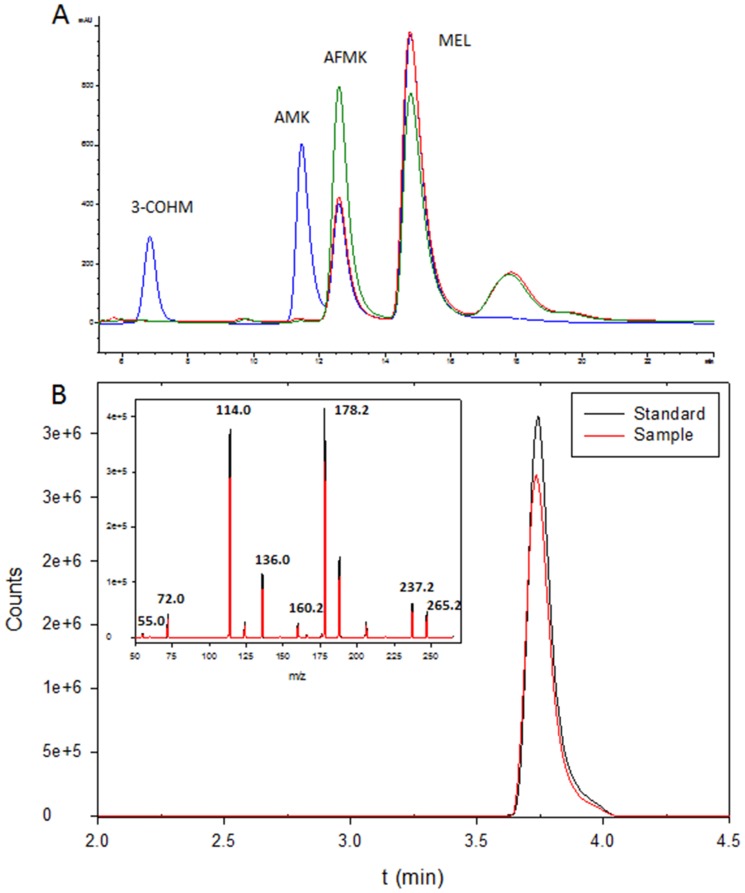
Presence of melatonin metabolites in DCFH_2_ photooxidation enhanced by melatonin. A) Chromatogram of standards of 3-COHM, AMK, AFMK and MEL (blue line), chromatogram of MEL (1 mM) with DCFH (10 µM) under light 5 min (red) or 10 min (green). B) Chromatogram and mass-spectrum obtained by HPLC-MS of AFMK standard (black) and AFMK present in sample (red) after MEL (1 mM) incubation with DCFH (10 µM) after 5 min of exposure to light.

Previous studies focused on photooxidation of MEL by protoporphyrin IX [40] or by 2-hydroxyquinoxaline [41] showed the presence of several kynureamines as metabolites. For this reason, N1-acetyl-N2-formyl-5-methoxykynuramine (AFMK), N1-acetyl-5-methoxykynuramine (AMK) or cyclic 3-hydroxymelatonin (3-COHM) were studied after DCFH_2_ exposure to light in the presence of MEL. When DCFH_2_ plus MEL was exposed for 30 seconds under light, we found a significant reduction of MEL concomitant with the presence of some new products. By comparing retention time as well as uv-spectrum with AFMK, AMK or 3-COHM standards, it was confirmed that AFMK was found after DCFH_2_ plus MEL were exposed to light (Fig. 5A). To ensure that AFMK is the compound generated in this reaction, a molecules produced and AFMK standard were compared by HPLC-MS obtained a positive confirmation of AFMK generation (Fig. 5B). The formation of AFMK requires the presence of two oxygen atom. Thus, when these experiments were performed in pure DMSO, DCF fluorescence was not found (data not shown).

### Dose response study of DCFH_2_ photooxidation by melatonin

**Figure 6:**
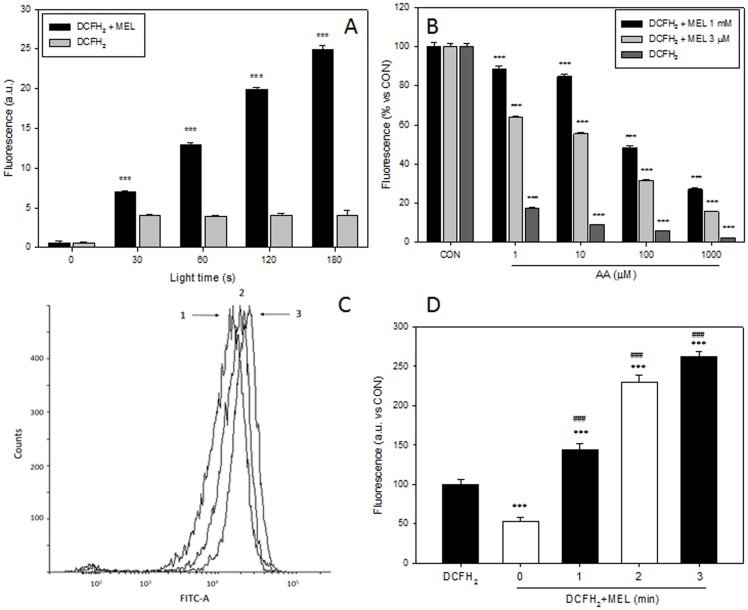
Dose dependence of DCFH_2_ in melatonin enhancement of photooxidation *in vitro* and in cellular models. A) Fluorescence of DCFH_2_ (0.1 µM) alone or plus MEL (3 µM) under light. B) Fluorescence of DCFH_2_ (0.1 µM) alone plus MEL (3 µM) or MEL (1 mM) in combination with AA (1–1000 µM) under light exposure for 60 seconds. C) Fluorescence, detected by flow cytometer, of HT22 cells incubated with 10 µM DCFH_2_ alone (1) or with 1 mM MEL plus 10 µM DCFH_2_ in darkness (2) or after 2 minutes of light exposure (3). D) Fluorescence, detected by microplate fluorimeter, of PC3 cells incubated with DCFH_2_ (10 µM) alone or with 1 mM MEL plus DCFH_2_ (10 µM) in darkness or after 1, 2 and 3 minutes of light exposure.

A dose response study was made by using 0.1 µM of DCFH_2_ and 3 µM MEL, the concentration of the indole found inside prostate LNCaP cells when they are incubated with 1 mM MEL for 6 hours [42]. Under these conditions, an increase of fluorescence was observed even after only 30 seconds (Fig. 6A). In addition, by using AA as antioxidant, there was a clear reduction in DCF formation also in a dose dependent manner (Fig. 6B). Furthermore a higher dose response study was done. So, in all MEL concentrations studied −1 nM to 1 mM- an increase in fluorescence was observed (Figure S2).

### DCFH_2_ photooxidation by MEL in culture cells

Prostate cancer (PC3) and hippocampal neuronal (HT22) cells were incubated with or without 1 mM MEL for 24 hours. Then, 10 µM of DCFH_2_-DA was added for 30 min prior to cytometer or fluorometric measurement. Those experimental conditions were chosen because there were normally employed by investigations describing pro-oxidant activity of the indoleamine [27], [43]–[45]. Changes in fluorescence among experimental groups were detected in both cell lines. Thus, when cells are incubated with MEL, a decrease in fluorescence is observed only when all experiment is performed in complete darkness (Fig. 6 C,D). When HT22 cells were exposed to light only for 1 minute, an increase of fluorescence and therefore DCF formation was observed. Same results were found in PC3 cells but light effect was lower. Thus, after 2 min under light an increase in fluorescence was also observed.

**Figure 7:**
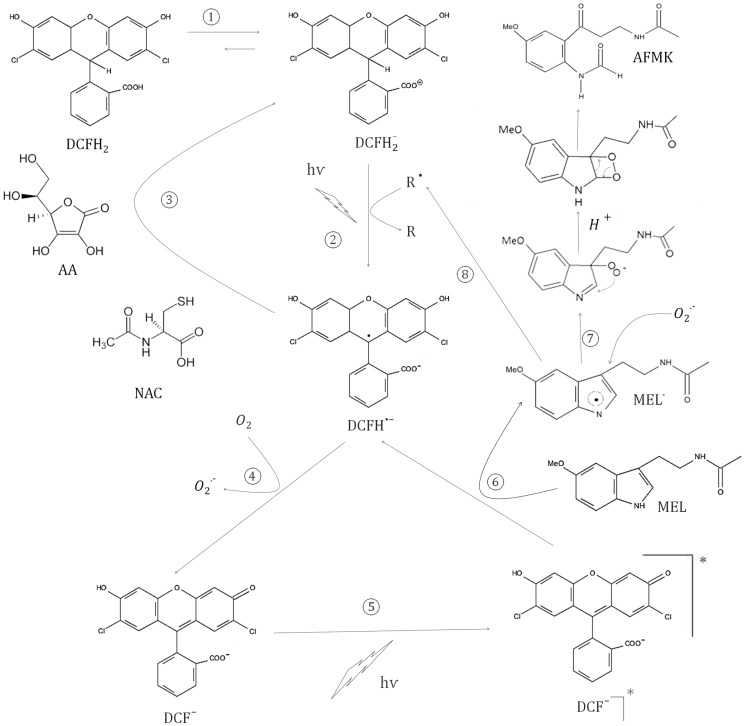
Diagram of proposal hypothesis about the mechanism of DCFH_2_, MEL and light reactions.

According to our results, a hypothetical pathway describing the potential reactions between DCFH_2_ and MEL are shown in Figure 7.

## Discussion

This study tried to understand an apparent dual role of MEL as pro-oxidant or anti-oxidant molecule. Mostly, the indolamine has been considered to scavenges free radicals or stimulates cell antioxidant defense [11], [17] while some reports described a pro-oxidant activity that in some context might induce cell death [27], [28]. The number of references that describe MEL as a pro-oxidant factor are considerably fewer that those describing antioxidant properties of the indole and also, few mechanistic explanations are proposed to explain its activity in promoting free radicals.

There is a clear controversy about the challenges and limitations of assay methods for measuring ROS [46]. In fact, some investigators considered essential to keep this limitations in mind for proper interpretation of data obtained [47]. Several reports have showed that DCFH_2_ is even oxidized in processes that do not actually involve ROS. Also, photo-irradiation incidental to spectrofluorometric or fluorescence microscopy observation has also been reported, therefore causing serious problems for the correct interpretation of DCFH_2_ as an indicator of ROS production [39]. For this reason and in order to evaluate the convenience of using DCFH_2_ in the evaluation of ROS production by melatonin, here it was performed an *in vitro* study about possible interactions between both, DFCH_2_-DA or DCFH_2_ and MEL, since those are probably the most widely employed probes for ROS analysis inside living cells.

Photo-oxidation of MEL has been previously reported in several occasions [40], [41], [48]. But while there was no increase in fluorescence when MEL was exposed to light alone in a free cell system, a clear increment was found when DCFH_2_-DA alone was exposed to light for a long time as previously described by others [38], [39], [49], [50].

The mechanism of DCFH_2_ oxidation is not clear yet [47], [51], [52]. In a previous report, Wrona et al. [53] have shown that a radical product DCFH^•−^ occur as an intermediate. DCFH^•−^ is necessary since its elimination by reaction with AA or NAC results in no DCF formation. Accordingly, when DCFH_2_ and MEL were incubated together in the absence of light, DCF was not detected, thus indicating that light is necessary for fluorescence enhancement.

On the other hand, high concentrations of DCFH_2_-DA (100 µM) and MEL (1 mM) are necessary to increase fluorescence in a cell-free system. Interestingly those experimental conditions are normally employed by investigations describing pro-oxidant activity of the indoleamine [27], [43]–[45]. This might explain the increment observed in DCF after MEL incubation under some situations without any net increase in ROS production. Our results prove this fact since antioxidants such as CAT or SOD are unable to inhibit DCF formation after MEL incubation. Furthermore, our results by using two different cell lines showed that under light, DCF assay might induce wrong in conclusions. Thus, MEL is inhibiting DCF formation when the experiment was performed in complete darkness but after a short exposition to light DCF fluorescence increase.

An accumulation of DCFH_2_ in V79 hamster cells after incubation with 10 µM of DCFH_2_ has been documented [46], [54]. Considering that we have used high concentrations of both, DCFH_2_ (10 µM) and MEL (1 mM) and the uptake of high concentrations of MEL might be compromised, being intracellular concentrations of the indole much lower than those applied in the culture media [55]. Here we studied the ability of MEL to increase DCF formation when employed at micromolar range concentration to assure that these observations were feasible to occur in the intracellular environment. In vitro experiments when MEL increases DCF fluorescence, high concentration of MEL (1 mM) in culture medium was used. For this reason, photooxidation of DCFH_2_ by MEL is possible as shown here.

Results obtained suggest that the mechanism by which DCF is produced from DCFH_2_ and DCFH_2_-DA is mechanistically different. As expected, these results confirmed that DCFH_2_ and DCFH_2_-DA are not the most adequate probes to test the ability of MEL to depurate free radicals in biological systems since fluorescence is a consequence of a side reaction that do not involve ROS participation. Also, considering mechanistic differences between DCFH_2_ and DCFH_2_-DA, it seems that DCFH_2_-DA could be a better choice since it is necessary a longer light exposure and a higher concentration to obtain less than 10 times of fluorescence when employed.

In conclusion, by using DCFH_2_ staining to measure redox control by MEL, it could be concluded than MEL might be a pro-oxidant molecule, while the real situation is very different since it is still working as an antioxidant compound and scavenging free radicals as shown in the diagram (Fig. 7). Most of the reactions shown in the depicted diagram (1–5) have already been demonstrated in previous reports. Thus, step 1 is due to physiological pH and step 2 was also previously described [53], [56]. By the effect of radical species or light, DCFH_2_^−^ is rapidly converted into DCFH^•−^ (2). AA and NAC acting as direct scavengers react with DCFH^•−^ (3) and inhibit DCF^−^ formation. DCF^−^ is generated from DCFH^•−^ when it reacts with oxygen to form superoxide (4). Under light, DCF^−^ absorbs energy and changes to the excited state DCF^−^]* (5) and MEL would be able to react with it to give DCFH^•−^ and MEL^•^ (6). This last reaction has been described when other molecules [57], such as GSH, are employed and it might be the reason why MEL is able to augment DCF fluorescence without increasing ROS production. Furthermore MEL^•^ can react with H_2_O, O_2_ or O_2_^•−^ to render AFMK (7). Other possibility is the role of this MEL^•^ as catalyst of the reaction 2 obtained MEL as product (R^•^ to R) (8). Thus, the increment in DCF production by MEL might not be a result of a pro-oxidant activity, but rather it seems that MEL is still working as an antioxidant in this context (6).

Altogether results presented here led us to propose that unless performed under dim red light all time of the performance of the assay, DCFH_2_ should not be employed for ROS measuring when working with melatonin since depending on time, DCFH_2_ or MEL concentration, it is possible to detect an increment in DCF^−^ fluorescence without any increment of ROS more on the contrary, while melatonin is still working as an antioxidant and a radical scavenger. Results published in the literature concerning pro-oxidant activity of melatonin in certain cell types should be re-evaluated, as this pro-oxidant action does not seem to be the underlying mechanism by which the indole induces cell death.

## Supporting Information

Figure S1 **Fluorescence spectrum (λ_exc_ = 480 nm, λ_em_ = 500–700 nm) of DCFH_2_-DA (100 µM) plus MEL (1 mM) under UV light (A), DCFH_2_ (10 µM) alone (C) or plus MEL (1 mM) under UV light at short times (B) or long times (D).**
(TIF)

Figure S2 **Fluorescence of DCFH_2_ (10 µM) plus several concentrations of MEL under light exposure (120 s).**
(TIF)
