# Supplementary figures and images for: Melatonin Enhances Photo-Oxidation of 2′,7′-Dichlorodihydrofluorescein by an Antioxidant Reaction That Renders N1-Acetyl-N2-Formyl-5-Methoxykynuramine (AFMK)
Source: PLoS One. 2014 Oct 2;9(10):e109257. doi: 10.1371/journal.pone.0109257 (PMC4183549; doi:10.1371/journal.pone.0109257)

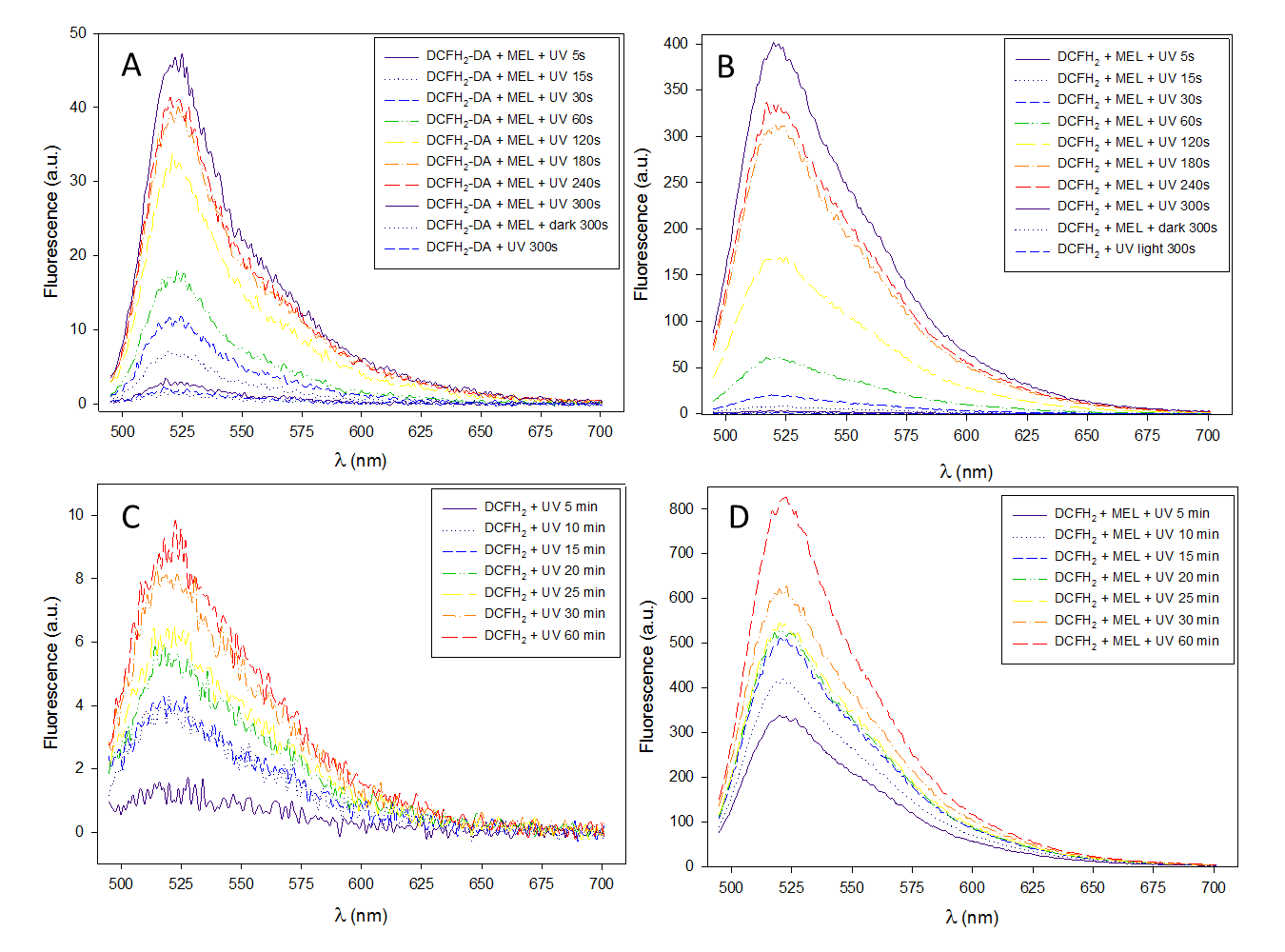

Supplement: Figure S1 — Fluorescence spectrum (λexc = 480 nm, λem = 500–700 nm) of DCFH2-DA (100 µM) plus MEL (1 mM) under UV light (A), DCFH2 (10 µM) alone (C) or plus MEL (1 mM) under UV light at short times (B) or long times (D). (TIF) [file pone.0109257.s001.tif]

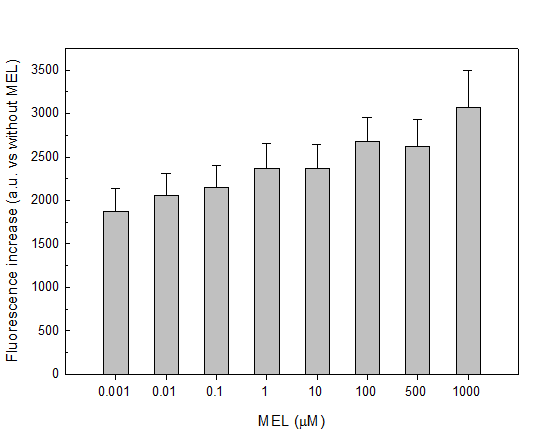

Supplement: Figure S2 — Fluorescence of DCFH2 (10 µM) plus several concentrations of MEL under light exposure (120 s). (TIF) [file pone.0109257.s002.tif]
